# Comparative Antidiabetic Activity of Aqueous, Ethanol, and Methanol Leaf Extracts of *Persea americana* and Their Effectiveness in Type 2 Diabetic Rats

**DOI:** 10.1155/2019/5984570

**Published:** 2019-10-16

**Authors:** N'Goran M. Kouamé, Camille Koffi, Kanga S. N'Zoué, N'Guessan A. R. Yao, Brahima Doukouré, Mamadou Kamagaté

**Affiliations:** ^1^Department of Clinical Pharmacology, UFR-SMB, Alassane Ouattara University, Bouaké, 01 BP V 18, Bouaké 01, Côte d'Ivoire; ^2^Department of Clinical Pharmacology, UFR-SMA, Félix Houphouët-Boigny University, Abidjan, 01 BP V 34, Abidjan 01, Côte d'Ivoire; ^3^Department of Pathological Anatomy, UFR-SMA, Félix Houphouët-Boigny University, Abidjan, 01 BP V 34, Abidjan 01, Côte d'Ivoire

## Abstract

Native to Mexico, *Persea americana* Mill. (avocado) is a fruit tree whose different parts (leaf, bark, roots, and stone) are used in traditional medicine especially against diabetes mellitus. The aim of this study was to investigate the beneficial effects of 28-day treatment with aqueous, ethanolic, and methanolic leaf extracts on glucose homeostasis in type 2 diabetic mellitus using Wistar rats. Type 2 diabetes was induced with nicotinamide (120 mg/kg, i.p.) and streptozotocin (65 mg/kg, i.p.). After 28 days of treatment, histopathological examination of the pancreas, kidneys, liver, and muscle (tibialis anterior) were realized. Biochemical markers were determined and an intestinal absorption test of D-glucose was performed. All extracts (100 mg/kg/day, p.o.) significantly (*p* < 0.001) reduced blood glucose level at the 28^th^ day of treatment with a more pronounced effect for methanolic extract. The treatments were well tolerated and induced a restoration of T-CHOL and HDL-C levels compared to the control group. Methanolic extract reduced the AIP (atherogenic index of plasma) by 45%. Histopathological analyzes of the pancreas showed regeneration of islets of Langerhans. Methanolic extract was the most effective in preventing intestinal glucose uptake up to 60.90% in relation to metformin. These results justify the use of this plant in traditional medicine against type 2 diabetes. However, other complementary studies should be done to identify the molecules responsible for this activity and their signaling voice.

## 1. Introduction

Diabetes mellitus is a metabolic disease caused by an insufficiency of insulin secretion associated or not with a bad use of this hormone by the body [1]. Once installed, this dysfunction causes a disruption of carbohydrate homeostasis (hyperglycemia), proteins, lipids, and electrolytes [2]. Unfortunately, 47.5% of people with diabetes are not screened. In the long run, this untreated metabolic pathology can lead to serious cardiovascular, neurological, and nephrological complications that are often fatal. In 2017, diabetes mellitus was the 7th leading cause of death and it killed 3.2 to 5 million humans in the world [3, 4].

In Africa, for economic and cultural reasons, 80% of the population uses medicinal plants from traditional medicine to treat themselves [5]. In the case of diabetes mellitus, approximately 800 plants have been identified and used alone or in combination in ethnomedicine as an antidiabetic treatment worldwide [6]. In African countries such as Côte d'Ivoire, Nigeria, Kenya, and Egypt, several ethnobotanical surveys confirmed that many of these medicinal plants are used against diabetes mellitus [7–9]. They include *Abizia harveyi*, *Ximenia americana*, *Eremophila maculata*, *Cola nitida*, *Punica granatum*, and *Persea americana* Mill. which showed potent activity against type 2 diabetes [10–15].

*Persea americana*, which is the subject of our study, is a tree native to the southeast of Mexico from where it has spread to all the tropical and subtropical regions of the world. It belongs to the class of Magnoliopsida and the Lauraceae family, which has more than 50 genera and about 3000 species [16, 17]. It is registered under No. 8845 in the national herbarium of Côte d'Ivoire. Ethnobotanical surveys have revealed that different parts of this plant (leaves, bark, fruit, and stone) are used alone or in combination with other plants against other diseases such as headache, rheumatism, dental pain, and skin diseases [18, 19].

Moreover, studies have shown antibacterial, anti-inflammatory, cardiovascular, antifungal, and antidiabetic activity of *P. americana*. With regard to diabetes, the work of Anitia et al. [20] and Lima et al. [21] revealed that aqueous and hydroethanolic extracts of *Persea americana* leaves have antihypoglycaemic activity on a streptozotocin- and alloxan-induced type 1 diabetic Wistar rat model. These studies demonstrated that this activity is due to the activation of the protein kinase B (PKB/Akt) pathway intracellularly [2, 20, 22].

However, there are little data on the antidiabetic activity of *P. americana* on type 2 diabetes although 90% of diabetes cases are type 2 according to the International Diabetes Federation [3]. In addition, the pathophysiology and risk factors of type 2 diabetes are different from those of type 1 diabetes. Obesity appears as one of the major risk factors. It would cause inflammatory reactions under the effect of hyperlipidemia leading to a decrease in the sensitivity of peripheral tissues to insulin, a phenomenon responsible for hyperglycemia [23, 24]. The first part of our study showed that the leaves of this plant seem quite tolerated [12].

The aim of this study work was to study the antidiabetic activity of aqueous, ethanolic, and methanolic extracts of *P. americana* leaves on a type 2 diabetes rat. Specifically, it was about evaluating and comparing the hypoglycaemic and hypolipidemic activity of these three extracts on the one hand. On the other hand, it was about to determine the therapeutic effects of these extracts on the islets of Langerhans and to evaluate their biotolerance at the hepatic, renal, and muscular level after a 28-day treatment. Finally, this work carries out a preliminary study of the mechanism of action of these extracts by measuring their capacity to prevent the intestinal absorption of glucose.

## 2. Materials and Methods

### 2.1. Chemical Kits and Reagents

Glibenclamide (Sanofi-Adventis, Daonil®, Paris, France), metformin (Denk pharma gmbH & Co KG, Metformin Denk®, Müchen, Germany), and D (+) glucose monohydrate (Riedel-de Haën, Berlin, Germany) were the chemicals used. Kits were obtained for determination of triglyceride (TG), total cholesterol (T-CHOL), high-density lipoprotein (HDL-C), blood urea nitrogen (BUN), creatinine (CREA), aspartate aminotransferase (AST), alanine aminotransferase (ALT), uric acid (UA), and alkaline phosphatase (AlkP) (Biolabo, Maizy, France). Packs for determining sodium (Na^+^), potassium (K^+^), calcium (Ca^2+^), and chloride (Cl^−^) (Socimed, Stains, France) were obtained. Compatible blood glucose test strips were obtained for the glucometer (Roche Diagnostics GmbH, Mannheim, Germany).

### 2.2. Experimental Animals

Male Wistar rats (*Rattus norvegicus var albinus*) of age 3 months, weighing 150–300 g, were obtained from the Department of Clinical Pharmacology of Félix Houphouët-Boigny University, Côte d'Ivoire. They were housed in polypropylene cages and acclimatized for 2 months prior to the experiments. The animals were kept under controlled conditions with temperature maintained at 25 ± 2°C, on light-controlled (12 h light/dark cycle) and free access to water and commercial chow (Faci, Abidjan, Côte d'Ivoire).

This study was approved by the Scientific Committee of the Training and Research Unit of Medical Sciences (UFR SM) of the University Félix Houphouët-Boigny (No. 2018/0014.5), and all procedures were performed in accordance with the NIH Guide for the Care and Use of Laboratory Animals [25].

### 2.3. Plant Material

The leaves of *P. americana* were collected before sunrise from Adiopodoumé (N 5° 19′ 3.49″ O 4° 8′ 8.66″), a village located at about 10 km from Abidjan (Côte d'Ivoire) in July 2018. The plant specimen was authenticated by the National Floristic Center of Félix Houphouët-Boigny University, and the voucher specimen was deposited in the herbarium under No. 8845. The dried leaves were powdered to obtain approximately 800 g.

### 2.4. Extraction

#### 2.4.1. Aqueous Extract (AE) of the Leaves of *Persea americana*

The dried powder of leaves was boiled in distilled water (5%, w/v) for 30 min, allowed to cool at room temperature, and filtered (Fisher Scientific, Fisherbrand®, New Hampshire, USA). The decoctions were lyophilized (Martin Christ, ALPHA 2-4 LDplus®, Osterode, Germany) and stored in the refrigerator at 2–4°C until used for the biological tests. The percentage yield based on the dried starting material of AE was 19.63% (w/w).

#### 2.4.2. Ethanol and Methanol Extracts (EE and ME) of the Leaves of *Persea americana*

The dried powder of leaves was extracted with absolute ethanol (10%, w/v) for 48 h by successive macerations three times. The ethanolic phases were pooled, the residue was removed by filtration, and then, the filtrate was dried at 40°C *in vacuo* (Memmert, Schwabach, Germany) to get a powder. The same extraction procedure was repeated for methanolic extract. The powder was stored in the dark at 4°C for subsequent experiments. The percentage yield for ethanolic and methanolic extracts based on the dried starting material was 15.42% and 18.35%, respectively.

### 2.5. Induction of Experimental Type 2 Diabetes Model (T2DM)

Streptozotocin (STZ) and nicotinamide (Sigma-Aldrich, St Louis, MO, USA) were used to damage the pancreatic *β*-cells [26, 27] and induced experimental T2DM according to the protocol of Masiello et al. [28] with slight modification. Intraperitoneal (i.p.) injection of freshly prepared STZ (65 mg/kg) mixed with 0.1 M citrate buffer (pH 4.5) in a volume of 1 ml/kg was performed in overnight fasted healthy male rats, 15 min after administration of nicotinamide (230 mg/kg, i.p.). Hyperglycemia was confirmed by polyuria, polydipsia, polyphagia, and elevation of blood glucose levels 72 h after the STZ-nicotinamide administration. After one week, rats that showed a fasting blood glucose level range of 200–300 mg/dl were considered T2DM and included in the study.

### 2.6. Selection of Doses

The selected dose (100 mg/kg/day, bw) of extracts (AE, EE, and ME) of *P. americana* was based on the preliminary study of their acute oral toxicity and hypoglycaemic activity of AE and ME [12]. Extracts were prepared in 2% aqueous Tween 80, considered as vehicle. Treatment was administered orally on a daily basis in a single dose for 28 consecutive days.

### 2.7. Evaluation of Antidiabetic Activity

The animals were grouped at random into six subgroups (5–7 rats per group):

  Group NDC (*n* = 5): nondiabetic control (NDC) that received only vehicle (10 ml/kg/day, bw)
  Group DC (*n* = 6): diabetic control (DC) that received only vehicle (10 ml/kg/days, bw)
  Group GLIB (*n* = 6): diabetic rats treated with glibenclamide (GLIB; 10 mg/kg/day, bw)
  Group AE (*n* = 7): diabetic rats treated with aqueous extract (AE; 100 mg/kg/day, bw)
  Group EE (*n* = 7): diabetic rats treated with ethanolic extract (EE; 100 mg/kg/day, bw)
  Group ME (*n* = 7): diabetic rats treated with methanolic extract (ME; 100 mg/kg/day, bw)

Fasting blood glucose level and body weight were recorded weekly, whilst food intake and water intake were monitored daily.

### 2.8. Biochemical Parameters

At the end of the 28^th^ day, animals were kept fasting overnight and euthanized by cervical dislocation under anesthesia isoflurane (Abbott, Forene®, Chicago, USA). Blood was collected in sterile vials, and serum was separated using a centrifuge at 1500 g for 10 min at 5°C (Jouan, BR4i®, Saint-Herblain, France) after blood coagulation, for the biochemical analysis. The following biochemical parameters were determined: BUN, UA, CREA, AST, ALT, AlkP, T-CHOL, HDL-C, TG, Ca^2+^, Na^+^, Cl^−^, and K^+^. Dosages were made using analyzer automation (Hitachi, 704R®, Tokyo, Japan) and electrolyte analyzer (SFRI, ISE 3000®, Gironde, France) with compatible reagent packs according to the manufacturer protocol. The blood glycaemia was estimated by the GOD-POD method using a glucometer (AccuChek® Active, Roche Diagnostics, Basel, Switzerland). The serum low-density lipoprotein cholesterol (LDL-C) was calculated by Friedwald [29] formula (1). Very low-density lipoprotein cholesterol (VLDL-C) was calculated based on equation (2) of Crook [30], and total lipids (T-LIP) was calculated based on formula (3) of Covaci et al. [31]. Atherogenic index of plasma (AIP) was calculated by using equation (3):

(1) $$\begin{array}{c} \text{VLDL}-\text{C}=\frac{\text{TG}}{2.2}, \end{array}$$

(2) $$\begin{array}{c} \text{T}-\text{LIP}=1.33\text{TG}+1.12\text{T}-\text{CHOL}+1.48, \end{array}$$

(3) $$\begin{array}{c} \text{AIP}=\frac{\text{LDL}-\text{C}}{\text{HDL}-\text{C}}. \end{array}$$

### 2.9. Histopathological Studies of Pancreas, Liver, Kidney, and Muscle

After euthanasia of rat and fasting blood collection, pancreas tissue, liver, muscle (tibialis anterior), and both kidneys were carefully removed, weighted, and then fixed in 10% buffered formalin. Histological preparations of these organs were performed in the pathological anatomy laboratory of the teaching hospital, Cocody (Côte d'Ivoire). Paraffin sections of 2–4 *μ*m were cut with microtome and stained with hematoxylin-eosin (HE), gomori trichrome, and Perls for microscopic examination (Motic® 1820, Hong Kong, China).

### 2.10. Intestinal Glucose Absorption Test

The intestinal glucose absorption test was performed according to Lima et al.'s protocol [21]. Normoglycaemic rats were sampled into 5 groups (*n* = 5). The fasting blood glucose level of each rat was determined at *t* = 0 h after overnight fasting (for 16 h) with free access to water. Groups 1 to 5 were treated orally with vehicle (10 ml/kg), metformin (15 mg/kg), AE (100 mg/kg), EE (100 mg/kg), and ME (100 mg/kg), respectively. After 30 min, D (+) glucose monohydrate (500 mg/kg, bw) was orally administered. Sixty minutes (60 min) later, all rats were euthanized in the similar condition described earlier on. Then, small intestines were carefully removed, and their contents were collected by perfusion of 50 ml distilled water. The content was centrifuged at 1500 g for 5 min, and the supernatant was used to determine glucose level based on the glucose dehydrogenase method using spectrophotometer.

### 2.11. Statistical Analysis

The results were presented as mean ± SEM. Then, statistical analysis of all the data obtained was evaluated using one-way ANOVA followed by the Newman–Keuls test (GraphPad Prism, version 5.01). The differences were considered as significant at *p* ≤ 0.05. Biochemical parameters variation (4) percentages were calculated as follows:

(4) $$\begin{array}{c} \text{variation}\left(\%\right)=\frac{\text{DCG}-\text{TGG}}{\text{DCG}}\times 100. \end{array}$$

where DCG: diabetic control glycaemia and TGG: test group glycaemia.

## 3. Results

### 3.1. Type 2 Diabetes Model (T2DM) from Rat

#### 3.1.1. Treatment Incidence on Glycaemia

**Figure 1:**
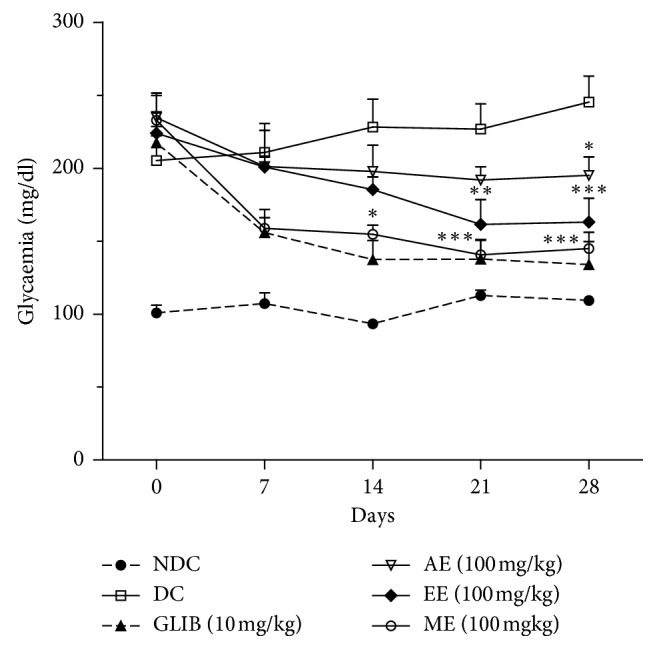
Effect of *P. americana* leaf extracts on glycaemia of type 2 diabetics rats. NDC: nondiabetic control treated with vehicle; DC: diabetic control treated with vehicle; GLIB: diabetic group treated with glibenclamide, 10 mg/kg; AE: diabetic group treated with aqueous extract, 100 mg/kg; EE: diabetic group treated with ethanol extract, 100 mg/kg; ME: diabetic group treated with methanol extract, 100 mg/kg. The values are expressed as mean ± SEM (*n* = 5–7). Statistically significant vs. DC: ^*∗*^*p* < 0.05; ^*∗∗*^*p* < 0.01; ^*∗∗∗*^*p* < 0.001.

The glycaemia regulation capacity of *P. americana* extracts on type 2 diabetes induced in Wistar rats treated during a 28-day period is shown in Figure 1. Before treatment, all diabetic rats had a hyperglycemia estimated at a mean value of 223 mg/dl. One week after starting the treatment, the average serum glucose of daily treated groups with *P. americana* AE, EE, and ME at a dose of 100 mg/kg was reduced at respective rates of 13.5, 14.3, and 31.8% in comparison with initial values, respectively. On the contrary, the untreated control group kept a constant and high glycaemia value of approximatively 210.8 mg/dl. At the end of the 28-day treatment period, the extracts brought about significant reduction of the glycaemia in diabetic rats with respective rates of 16.3%, 20.8%, and 37.4% for AE, EE, and ME. These results proved all three extracts to have regulated type 2 diabetes in rats as compared to the untreated control group whose glycaemia remained elevated. However, extract ME seemed to be the more active than glibenclamide. This extract reduced average glycaemia of diabetic rats at 145 mg/dl, while glibenclamide at 133.8 mg/dl.

#### 3.1.2. Incidence of Treatment on T2DM Rats' Nutritional State

**Figure 2:**
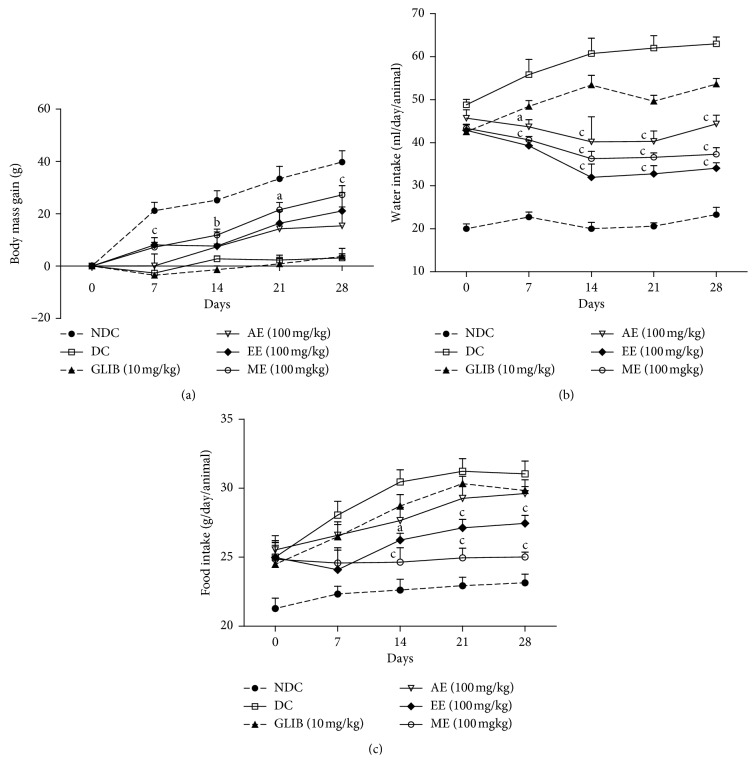
Effect of *P. americana* leaf extracts on body mass gain (a), water (b), and food intake (c) of type 2 diabetic rats. NDC: nondiabetic control treated with vehicle; DC: diabetic control treated with vehicle; GLIB: group treated with glibenclamide, 10 mg/kg; AE: rats treated with aqueous extract, 100 mg/kg; EE: rats treated with ethanol extract, 100 mg/kg; ME: rats treated with methanol extract, 100 mg/kg. The values are expressed as mean ± SEM (*n* = 5–7). (a) Significantly different vs. DC; (b) Significantly different vs. GLIB; (c) Significantly different vs. DC and GLIB.

The influence of the administration of *P. americana* extracts on body weight, water uptake, and food uptake is present in Figure 2. After the 28-day treatment period, all diabetic rats treated with AE, EE, and ME at a daily dose of 100 mg/kg showed an increase in body weight, whereas the weights of DC and GLIB groups remained the same (see Figure 2(a)). The respective increase rate in body weight of AE, EE, and ME reached 7.61, 10.52, and 15.22%. Thus, the weight gain has been significant for rats fed with ME since the first week of treatment. Although the rise of body weight of treated rats was significantly superior to that of the DC group (not treated), it was significantly inferior to the average of normoglycaemic rats (NDC group). The increase in average was 16.64% (39.8 g) consecutive to the 28 days treatment period.

As for the daily feeding during experiment, data collected (Figure 2(b)) showed that T2DM rats, which received each extract dose of 100 mg/kg/day, took lesser food than the untreated control group (DC). This polyphagia was slighter for groups EE and ME than that in groups DC and GLIB.

Figure 2(c) shows variation of daily consumption of water per rat. From the first week up to the 14^th^ treatment day, rat groups AE, EE, and ME had significantly reduced polydipsia at 12.04, 25.64, and 16.74%, respectively, which was maintained between 31.9 and 44 ml/rat/day, until the 28^th^ day. In contrast, water uptake by DC and GLIB groups increased progressively to reach respective values of 53 and 63.4 ml/rat/day. As for the NDC group, its average water consumption was 20.66 ml/rat/day.

#### 3.1.3. Organ to Body Weight Ratio

After the 28-day treatment, kidney to body weight ratios of T2DM rats from DC and GLIB groups were significantly (*p* < 0.01) higher than that of normoglycaemic animals (NDC).

Additionally, the liver to body weight ratio significantly increased for the group treated with glibenclamide. For groups treated with the different extracts, the ratios were within the range of normal values (Table 1).

### 3.2. Analysis of Biochemical Parameters

#### 3.2.1. Lipidic Profile

**Figure 3:**
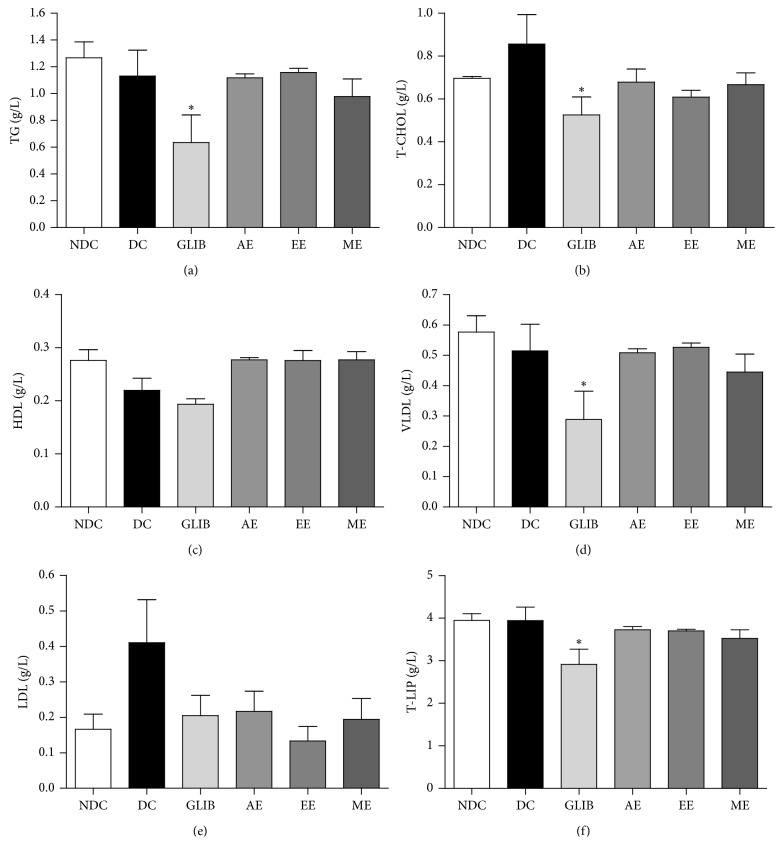
Effect of *P. americana* leaf extracts on triglyceride (a), total cholesterol (b), high (c), very low (d), and low (e) density lipoprotein and total lipids (f) of type 2 diabetic rats after 28 consecutive days of treatment. NDC: nondiabetic control group treated with vehicle; DC: diabetic control group treated with vehicle; GLIB: group treated with glibenclamide, 10 mg/kg; AE: group treated with aqueous extract, 100 mg/kg; EE: group treated with ethanol extract, 100 mg/kg; ME: group treated with methanol extract, 100 mg/kg. The values are expressed as mean ± SD. Statistically significant vs. NDC (*n* = 5–7): ^*∗*^*p* < 0.05.

The lipidic profile of the animals set on day 28 of the experiment is presented in Figure 3. Glibenclamide (10 mg/kg/day) reduced serum lipidic charges. This decreased rate in T-CHOL, T-LIP, VLDL-C, and TG was significant with respective values of 38.7, 26.2, 43.9, and 43.9% in relation to the DC group. In addition, AE, EE, and ME at a dose of 100 mg/kg/day were able to restore proportion of T-CHOL and HDL-C at a level comparable to that of the NDC group (0.70 g/L and 0.28 g/L). Yet, rates of TG, VLDL-C, and T-LIP were slightly below NDC group's normal values. The LDL-C and the AIP were lower in groups treated with the extracts than in untreated diabetic groups (DC). These decreases reached respective rates of 67.5% and 45.0% for groups EE and ME in the case of LDL-C.

#### 3.2.2. Transaminasemia and Blood Ionogram Parameters

Table 2 presents the hepatic, renal function markers, and ionogram of T2DM rats after the 28 days of treatment. Before the treatment, all diabetic rats had higher indication of liver and kidney failure than the NDC group. Ethanolic and methanolic extracts induced nonsignificant decrease in liver and tissue markers (AST, ALT, and AlkP) from 7.4 to 47.2% compared to the DC group. Concerning with AE group, the AST proportions were reduced to 12.6%. For renal function markers, the BUN level was down 34.4% in the AE group. As for markers of ethanolic and methanolic extracts, they decreased 25.1% for BUN and 57.1% for UA. As for CREA, it was reduced in diabetic groups with a decrease rate of 3.4% and 17.5% for AE and GLIB groups, respectively.

Blood ionogram showed, in general, that proportions of Na^+^, Cl^−^, and Ca^2+^ did not vary significantly after the 28 days of treatment period. Meanwhile, K^+^ values were reduced 13.5% in group EE in relation to the control group (DC).

### 3.3. Histopathological Analyses

**Figure 4:**
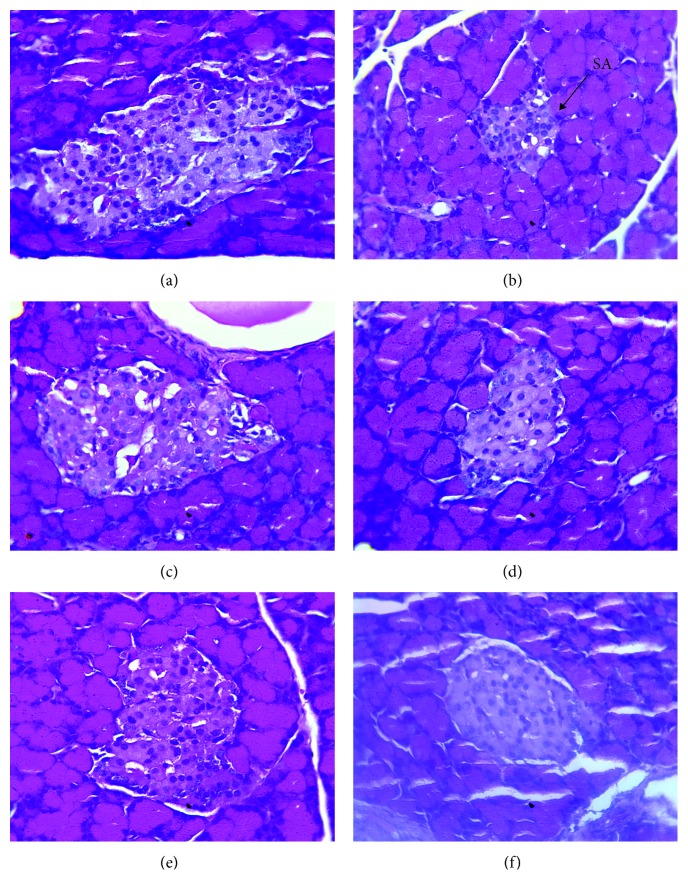
Effect of *P. americana* leaf extracts on islets of Langerhans of type 2 diabetic rats after 28 consecutive days of treatment. Pancreas tissue section stained with hematoxylin-eosin (×400). (a) Nondiabetic control (NDC), normal islet; (b) diabetic control (DC), severe atrophy islet (SA); (c) GLIB (10 mg/kg), mild atrophy islet diameter composed of a small number of hyperchromatic nuclei; (d–f) diabetic rats treated, respectively, with AE, EE, and ME (100 mg/kg) extracts, regeneration of islets of Langerhans.

Histopathological preparations of pancreatic tissue of rats are presented in Figure 4. Observation of histopathological sections of NDC rats group showed several islets of Langerhans with normal architecture (Figure 4(a)). However, a severe atrophy of pancreatic islets and a reduction in the number of cells due to type 2 diabetes appeared in control diabetic rats (Figure 4(b)). In rats of the GLIB group, this atrophy was slight (Figure 4(c)). But, islets cells in diabetic rats treated with AE, EE, and ME during the 28 days were recovered partially (Figures 4(d)–4(f)).

**Figure 5:**
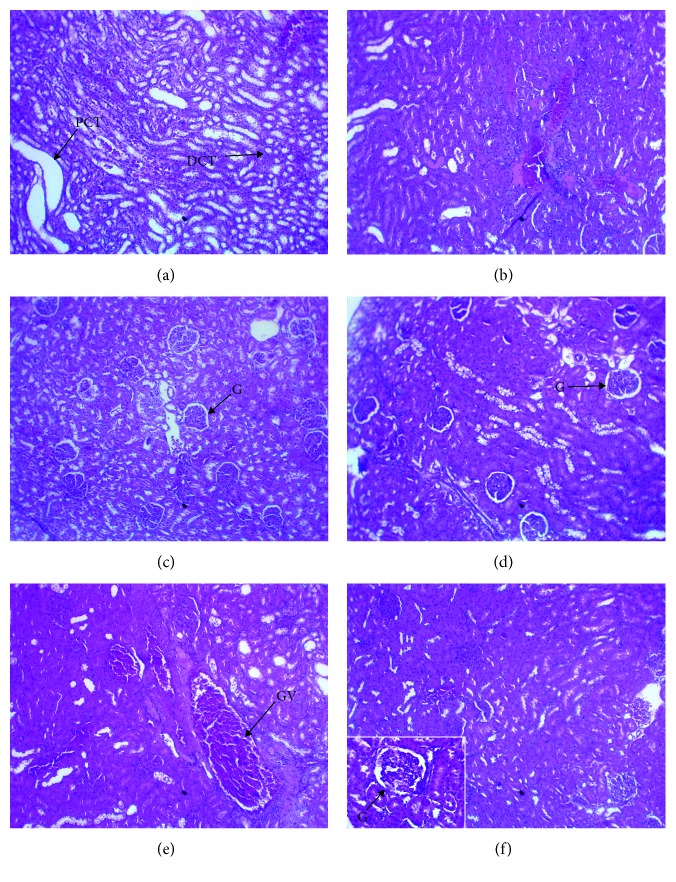
Effect of *P. americana* leaf extracts on kidney of type 2 diabetic rats after 28 consecutive days of treatment. Kidney tissue section stained with hematoxylin-eosin (×250 or 400). (a) Nondiabetic control (NDC), kidney tissue has distal and proximal convoluted tubule (DCT and PCT) of normal architecture. (b) Diabetic control (DC): kidney tissue without histological abnormality. (c) GLIB (10 mg/kg), renal tissues with glomeruli (G) normal appearance. (d–f) Diabetic rats treated, respectively, with AE, EE, and ME (100 mg/kg), kidney tissue without architectural anomaly with the presence of congestive vessel (*GV*).

The kidney tissue showed a normal appearance with glomeruli located in the renal cortex zone surrounded by a clear space (Bowman space) and apparent proximal and distal convoluted tubules (Figure 5).

**Figure 6:**
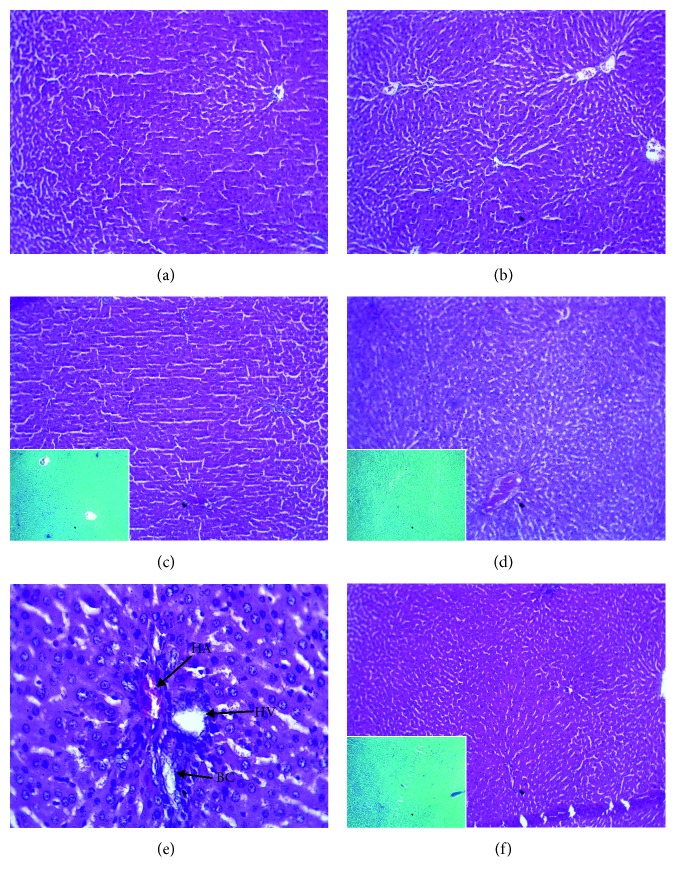
Effect of *P. americana* leaf extracts on the liver of type 2 diabetic rats after 28 consecutive days of treatment. Liver tissue section stained with hematoxylin-eosin and Masson (×250 or 400). (a) Nondiabetic control (NDC), liver tissue no irregularity. (b) Diabetic control (DC), representative picture shows hepatocyte regular spans architecture without histological abnormality. (c) GLIB (10 mg/kg), normal liver architecture without fibrous lesion; (d–f) Diabetic rats treated, respectively, with AE, EE, and ME (100 mg/kg), hepatic parenchyma of normal architecture with a portal space compound of the hepatic artery (HA), hepatic vein (HV), and bile canaliculus (BC).

The liver tissues showed hepatic lobules with regular and normal hepatic cells. There was no lymphocytal infiltration. The paraffin sections stained with Masson trichrome and Perls did not reveal neither fibrosis nor hemosiderinic deposits (Figure 6).

**Figure 7:**
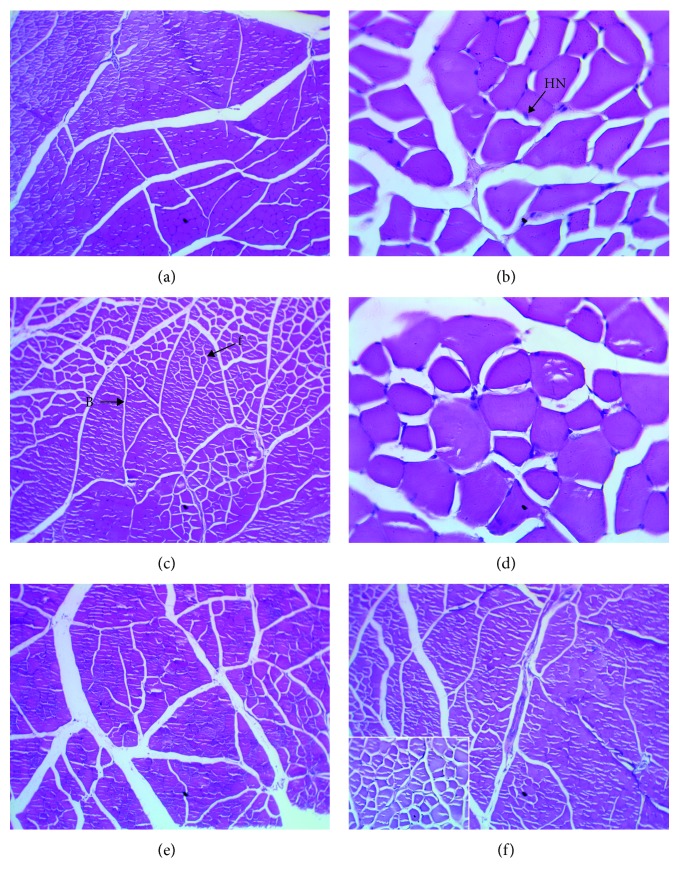
Effect of *P. americana* leaf extracts on muscle of type 2 diabetic rats after 28 consecutive days of treatment. Representative muscle tissue section stained with hematoxylin-eosin (×250 or 400). (a) Nondiabetic control (NDC), muscle tissue composed of bundles of normal aspects. (b) Diabetic control (DC), tissue muscle presents cells muscle without abnormal histological with hyperchromatic nuclei (*HN*). (c) GLIB (10 mg/kg), representative tissue bundles (B) and fibers (f) normal muscle architecture; (d–f) Diabetic rats treated respectively with AE, EE, and ME (100 mg/kg), muscle cell showing no histological abnormality.

Transversal histological sections of skeletal muscle tibialis anterior observed by lower (×250) and higher (×400) increments showed that muscular fibers with cells were made up of nuclei without cytological atypia (Figure 7).

### 3.4. Intestinal Absorption of Glucose in Normoglycaemic Rats

**Figure 8:**
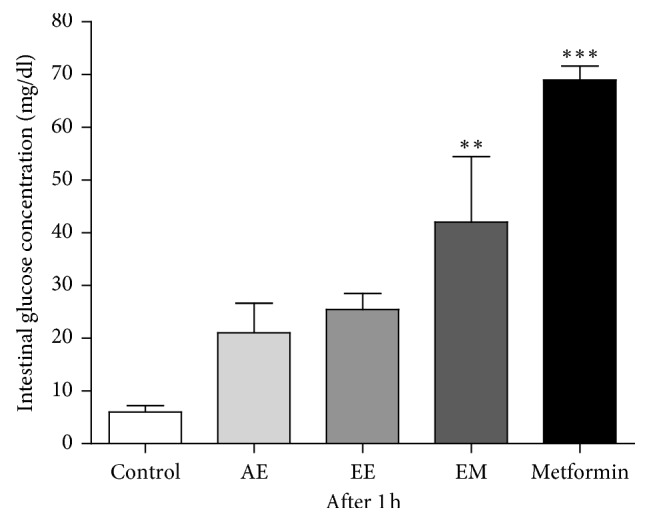
Effect of *P. americana* leaf extracts on intestinal glucose absorption in normoglycaemic rats. Control: group treated with vehicle; AE: group treated with aqueous extract, 100 mg/kg; EE: group treated with ethanol extract, 100 mg/kg; ME: group treated with methanol extract, 100 mg/kg; metformin: group treated with metformin, 15 mg/kg. The results are expressed as mean ± SEM. (*n* = 5). Statistically significant vs control: ^*∗∗*^*p* < 0.01; ^*∗∗∗*^*p* < 0.001.

The results of the intestinal absorption of glucose in normoglycaemic rats are shown in Figure 8. These results showed an intestinal glucose depletion in normoglycaemic rats with prior AE, EE, and ME administration (30 min) at a dose of 100 mg/kg. The respective rates obtained were 30.45, 36.83, and 60.90% compared with positive control (metformin). Precisely, only the ME inhibited significantly (*p* < 0.01) glucose absorption in contrast with the negative control. However, this hypoglycaemic activity of ME is weaker than that of the reference compounds (metformin).

## 4. Discussion

The study of the antidiabetic activity of the aqueous, ethanolic, and methanolic extracts of *P. americana* leaves administered at a dose of 100 mg/kg/day for 28 days confirmed the ability of these extracts to help the organs of diabetic animals to regulate glucose metabolism in type 2 diabetes. The fasting hyperglycemia initially between 200 and 300 mg/dL has fallen to 195.1 mg/dL for the AE group, 163.1 mg/dL for the EE group, and 145 mg/dL for the ME group after 28 days of treatment, whereas the diabetic control (DC) group maintained high fasting hyperglycemia at 245.3 mg/dL. This reduction in blood glucose was significant as early as the second week of treatment in the case of ethanolic and methanolic extracts. This early ability of *P. americana* extracts to regulate fasting glucose levels in diabetic animals was highlighted by the works of Brai and Lima [21, 32]. In effect, this efficacy is reflected clinically by the significant improvement of hyperglycemia-related symptoms of polyuria, polyphagia, and polydipsia in treated rats compared to NDC rats. Thus, this glycaemic reduction explains the limitation of the weight loss of treated diabetic rats in relation to the NDC rats and unlike glibenclamide, which would lead to a severe weight loss [21]. This improvement in clinical symptoms that characterized type 2 diabetes had already been reported in the work of Oliveira and Lima et al. [21, 33].

This ability of organic extracts, in particular the methanolic extract, to regulate blood glucose levels has already been demonstrated with several African pharmacopoeia plants, such as *Albizia harveyi*, *Ximenia americana*, *Eremophila maculata*, *Cola nitida*, *and Punica granatum* [10, 11, 13–15]. This potential could be explained by the composition of these extracts in bioactive phytochemical molecules, especially in polyphenols. Indeed, a phytochemical study carried out previously had highlighted the presence of polyphenols, flavonoids, saponins, alkaloids, tannins, sterols, terpenes, and coumarins in these extracts. The concentration of these polyphenol extracts was 2707.3 ± 155.4, 2952.7 ± 166.0, and 1873.1 ± 63.5 (GAE) *μ*g/g of extract, respectively, for AE, EE, and ME [12]. These secondary metabolites have the ability to regulate blood glucose through several signaling pathways. Saponins, polyphenols, and, especially, flavonoids have a hypoglycemic activity by inhibition of intestinal absorption of glucose and glycogenolysis, restoring beta-cells' integrity and enhancing insulin release. Inhibition of the activity of several enzymes such as *α*-amylase, *α*-glucosidase, and glucose-6-phosphatase (G6Pase) would lead to reduction of glucose bioavailability [34–37]. Thus, these molecules would increase the peripheral use of glucose by stimulating translocation of GLUT4 in skeletal muscle [38].

The extracts have an effect comparable to that of metformin at the intestinal level. The extracts of *P. americana* were able to inhibit the intestinal absorption of glucose, in particular the methanolic extract whose inhibitory activity represented 60.9% of that of metformin. The half-maximal inhibitory concentration (IC_50_) of the methanolic extract of *P. americana* leaves was 0.219 ± 0.012 mg/mL for *α*-amylase and 0.067 ± 0.001 mg/mL for *α*-glycosidase [39]. The work of Kim et al. [37] has confirmed this hypothesis by showing its ability to inhibit the activity of the intestinal glucose transporter SGLT1 too. The present study shows that extracts of *P. americana* would act via insulin. In fact, the islets of Langerhans, which had been destroyed by streptozotocin and nicotinamide, were regenerated under the extracts (see Figure 4). This regeneration or islet protection was observed with the 28-day treatment with a hydroethanolic extract of *P. americana* leaves at 300 mg/kg/day in type 1 diabetic rats [21]. This confirms its usefulness not only in type 1 diabetes but also in type 2 diabetes.

The study of the lipid profile of rats at the end of the treatment period showed that extracts of *P. americana* were generally lipid lowering. Indeed, the effects of glibenclamide on T-LIP, TG, T-CHOL, and VLDL-C are comparable to those of the extracts. Moreover, the extracts lead to a fall in the LDL-C level and the AIP (LDL/HDL) and an increase in HDL-C. Therefore, these extracts would be antiatherogenic and would help reduce the phenomenon of insulin resistance by regulating lipid homeostasis [40]. This hypocholesterolemic effect was also observed in Brai's work in 2007, on normoglycemic rats on a hypercholesterolemic diet [32]. Postprandial hyperglycemia and elevated LDL/HDL ratio in type 2 diabetes are risk factors for the occurrence of cardiovascular complications (microangiopathies and macroangiopathies) such as arteriosclerosis and retinopathy.

These results confirm that the extracts have comparable effects to metformin by inhibiting postprandial hyperglycemia, on the one hand, and antihyperglycemic and hypolipidemic effects similar to glibenclamide. On the other hand, these extracts would reduce the risk of cardiovascular complications by significantly reducing the atherogenic index of plasma. Another advantage is that the first part of this study had shown that these extracts would be nonhypoglycemic in contrast to glibenclamide. In addition, it seems well tolerated (LD50 ≥ 5000 mg/kg) [12]. The ratio of kidney or liver mass of rats to body mass of treated diabetic animals was comparable to that of healthy rats (NDC). It is different from that of the DC group and GLIB group, which had a significantly high ratio (see Table 1). The results suggest that the extracts of this plant would have protected the kidneys and liver of diabetic rats from complications related to type 2 diabetes. This nephroprotective and hepatoprotective activity of *P. americana* extracts is attributable to alkaloids, flavonoids, tannins and saponins that would have an antioxidant, vaxorelaxant, bradycardic, and hypotensive property. In addition, these extracts would improve glomerular filtration [22, 41–44]. This ability of *P. americana* extracts to protect the kidneys and liver is confirmed by the enhancement of tissue, liver, and kidney markers except AE that seems toxic to the liver and kidneys. This biotolerance of *P. americana* has been reported by the work of Kamagaté et al. [12, 45, 46], which showed that the ethanolic and methanolic extracts of this plant had a cytoprotective effect against the toxicity induced by the high dose of paracetamol (2000 mg/kg, p.o.) and streptozotocin (50 mg/kg, i.p.). Histological analyzes of the liver, kidneys, and muscle (tibialis anterior) would confirm this biotolerance by revealing no abnormalities visible under the light microscope.

## 5. Conclusion

The present study showed that *P. americana* is able to help organs restore glucose and lipid homeostasis, particularly in the case of type 2 diabetes while being well tolerated. This justifies the use of this plant in traditional medicine to treat diabetes mellitus and avoid the complications generated by this disease.

However, it would be important to conduct further studies to elucidate its mechanism of action and evaluate its long-term toxicity in order to popularize its use.

## Figures and Tables

**Table 1:** Organ to body weight ratio percentages for type 2 diabetic model rats treated for 28 consecutive days.

Ratio (%)	Groups					
	NDC (*n* = 5)	DC (*n* = 6)	GLIB (10 mg/kg) (*n* = 6)	AE (100 mg/kg) (*n* = 7)	EE (100 mg/kg) (*n* = 7)	ME (100 mg/kg) (*n* = 7)
Ratio kidney	0.550 ± 0.015	0.790 ± 0.041^*∗∗*^	0.730 ± 0.065^*∗*^	0.644 ± 0.042	0.577 ± 0.041	0.549 ± 0.011
Ratio liver	2.70 ± 0.070	3.78 ± 0.183^*∗∗*^	3.15 ± 0.178	3.47 ± 0.250	2.97 ± 0.229	2.786 ± 0.051

NDC: nondiabetic control group treated with vehicle; DC: diabetic control group treated with vehicle; GLIB: group treated with glibenclamide, 10 mg/kg; AE: group treated with aqueous extract, 100 mg/kg; EE: group treated with ethanol extract, 100 mg/kg; ME: group treated with methanol extract, 100 mg/kg. The values are expressed as mean ± SEM (*n* = 5–7). Statistically significant vs. NDC: ^*∗*^*p* < 0.05; ^*∗∗*^*p* < 0.01.

**Table 2:** Biochemical parameters of type 2 diabetic rats after 28 consecutive days of treatment.

Biochemical parameters	Groups									
	NDC (*n* = 5)	DC (*n* = 6)	GLIB (10 mg/kg) (*n* = 6)	Variation (%)	AE (100 mg/kg) (*n* = 7)	Variation (%)	EE (100 mg/kg) (*n* = 7)	Variation (%)	ME (100 mg/kg) (*n* = 7)	Variation (%)
AST (U/L)	172.52 ± 18.38	477.18 ± 144.23	506.08 ± 112.20	6.1	300.70 ± 111.19	−12.6	410.23 ± 115.14	−14.0	448.46 ± 134.45	−31.0
ALT (U/L)	87.84 ± 17.49	162.35 ± 33.88	104.37 ± 13.67	−35.7	209.11 ± 64.33	28.8	142.14 ± 35.45	−12.4	150.257 ± 33.90	−7.4
AlkP (U/L)	389.28 ± 30.96	838.78 ± 196.46	642.88 ± 226.23	−23.4	940.69 ± 176.21	12.1	524.16 ± 170.58	−37.5	443.29 ± 51.12	−47.2
BUN (mg/L)	29.40 ± 1.97	66.00 ± 12.42	65.17 ± 18.52	−1.3	43.29 ± 7.95	−34.4	32.86 ± 5.30	−50.2	28.29 ± 1.64	−57.1
CREA (mg/L)	2.83 ± 0.67	4.18 ± 0.19	3.45 ± 0.35	−17.5	4.04 ± 0.25	−3.4	3.49 ± 0.10	−16.7	3.67 ± 0.15	−12.2
UA (mg/L)	22.32 ± 1.71	26.67 ± 4.46	31.05 ± 3.43	16.4	33.29 ± 7.27	24.8	19.99 ± 2.71	−25.1	27.39 ± 2.89	2.7
Na^+^ (mmol/L)	143.40 ± 0.51	143.17 ± 1.33	136.00 ± 0.63	−5.0	143.43 ± 1.63	0.2	142.39 ± 1.14	−0.5	145.43 ± 1.17	1.6
K^+^ (mmol/L)	6.48 ± 0.49	7.29 ± 0.57	7.41 ± 0.24	1.7	7.23 ± 1.00	−0.8	6.30 ± 0.23	−13.5	7.27 ± 0.45	−0.2
Cl^−^ (mmol/L)	103.20 ± 1.53	103.33 ± 0.88	100.33 ± 0.42	−2.9	102.86 ± 1.10	−0.5	103.44 ± 1.27	0.1	107.57 ± 1.41	4.1
Ca^2+^ (mg/L)	104.36 ± 2.21	99.65 ± 5.39	95.13 ± 5.54	−4.5	102.79 ± 4.35	3.1	108.34 ± 2.92	8.7	111.41 ± 2.52	11.8
AIP (LDL/HDL)	0.673 ± 0.239	1.786 ± 0.395	1.121 ± 0.342	−43.2	0.783 ± 0.202	−26.2	0.542 ± 0.175^*∗*^	−38.1	0.747 ± 0.259^*∗*^	−45.0

AST: aspartate aminotransferase; ALT: alanine aminotransferase; BUN: blood urea nitrogen; AlkP: alkaline phosphatase, CREA: creatinine; UA: uric acid; Na^+^: sodium ion; K^+^: potassium ion; Cl^−^: chloride ion, Ca^2+^: calcium ion, AIP: atherogenic index of plasma. NDC: nondiabetic Control treated with vehicle; DC: diabetic control treated with vehicle; GLIB: group treated with glibenclamide, 10 mg/kg; AE: group treated with aqueous extract, 100 mg/kg; EE: group treated with ethanol extract, 100 mg/kg; ME: group treated with methanol extract, 100 mg/kg. The values are expressed as mean ± SEM (*n* = 5–7). ^*∗*^Statistical significance vs. DC (*p* < 0.05).

## Data Availability

The data used to support the findings of this study are included within the article.
